# Analogy of silicon and boron in plant nutrition

**DOI:** 10.3389/fpls.2024.1353706

**Published:** 2024-02-06

**Authors:** Huachun Sheng, Yuyan Lei, Jing Wei, Zhengming Yang, Lianxin Peng, Wenbing Li, Yuan Liu

**Affiliations:** ^1^Sichuan Provincial Qiang-Yi Medicinal Resources Protection and Utilization Technology and Engineering Laboratory, Southwest Minzu University, Chengdu, Sichuan, China; ^2^Tibetan Plateau Ethnic Medicinal Resources Protection and Utilization Key Laboratory of National Ethnic Affairs Commission of the People’s Republic of China, Southwest Minzu University, Chengdu, Sichuan, China; ^3^Key Laboratory of Coarse Cereal Processing of Ministry of Agriculture and Rural Affairs, Chengdu University, Chengdu, Sichuan, China

**Keywords:** silicon, boron, cell wall, crosslinking, structural function

## Abstract

Silicon (Si) and boron (B) are a class of elements called metalloids, which have properties like metals and non-metals. Si is classified as a quasi-essential element, while B is a micronutrient element for plants. Nowadays, numerous discoveries have shown the analogy of silicon and boron in plant nutrition. In this minireview, the molecular mechanisms for the transport of these two metalloids are compared. We also discussed the chemical forms of Si and B and their functional similarity in response to environmental stresses in plants. In conclusion, it can be proposed that cell wall-bound silicon rather than silica might partially replace boron for plant growth, development, and stress responses, and the underlying mechanism is the Si contribution to B in its structural function.

## 1 Introduction

Plants absorb the mineral nutrients from the soil to satisfy the demand for growth and survival. Among the mineral elements, the metalloid of Si is not considered an essential element for plants, because there is no evidence for the involvement of Si in metabolism (Epstein, 1999). However, Si alleviation of environmental stresses (including various biotic and abiotic stresses) has been observed in several plant species by comparing between plants with and without the application of Si fertilizers (Coskun et al., 2019; de Tombeur et al., 2023). In contrast, another metalloid B mainly exists in the cell wall of plants and has been listed as an essential element due to its crosslink with the pectic polysaccharides of rhamnogalacturonan II (RG-II) (O'Neill et al., 2004). The deficiency of micronutrient B results in the inhibition of young leaf expansion, root elongation, and fertility (Onuh and Miwa, 2021), while high concentrations of B are toxic to plants and cause the relaxed chromatins and DNA double-strand breaks in root meristems (Sakamoto et al., 2018). Therefore, the maintenance of B homeostasis is necessary for plant growth and development.

It has been reported that a supply of Si can alleviate the symptoms induced by B disorder in plants (Pavlovic et al., 2021). Indeed, Si alleviation of B toxicity was reported in many plants, including rice, cotton, wheat, barley and others (Gunes et al., 2007a; Gunes et al., 2007b; Inal et al., 2009; Chen et al., 2019; de Souza Junior et al., 2019; Savic et al., 2023). The formerly suggested common mechanism is that silicic acid interacts with the boron acid in soil solution and inside the plant roots to form Si-B complexes, leading to the B immobilization and reduced uptake of B (Inal et al., 2009). Moreover, Si raises the level of *BOR2* gene expression, whose protein product serves as an efflux transporter for B extrusion, thereby contributing to B detoxification in the apoplast (Akcay and Erkan, 2016). Very recently, Savic et al. (2023) found that Si can enhance the tolerance of B toxicity by the extension of the cell wall binding sites for B in the Si-accumulating species of wheat, but not in the Si-non-accumulating plant of sunflower. These findings imply the complicated roles of Si in B toxicity responses in different plants.

In addition to improving plant growth under B toxicity, several researchers have shown that Si can significantly improve the growth of rapeseed plants under B limitation (Liang and Shen, 1994; Réthoré et al., 2023), and the underlying mechanisms are that (1) the expression of *BnaNIP5;1* and *BnaBOR1;2c* genes are up-regulated by Si in both young leaves and roots for B uptake; (2) Si could induce the remobilization of previously fixed B from old leaves to young tissues. Interestingly, Si doesn’t alter the B concentration in leaves but increases the biomass of leaves in B-deficient rapeseed plants (Réthoré et al., 2023), suggesting that Si has a similar role to B in leaf expansion. Combined with the discovery of Si-pectin complexes in plants (Sheng and Chen, 2020), it was speculated that Si may partially replace B for plant growth and development because of the analogy between them. In this minireview, we analyze the similarities of their transport system and chemical forms, as well as their roles in stress response, to investigate this question.

## 2 Transport system of Si and B in plants

Both Si and B are taken up by plants in the form of noncharged molecules, H_4_SiO_4_ and H_3_BO_3_. Their transports are primarily governed by a cooperative system that consists of influx and efflux transporters (Takano et al., 2008; Ma and Yamaji, 2015). The influx transporters of Si and B belong to the Nodulin 26-like intrinsic proteins (NIPs) subfamily in the aquaporin family, while efflux transporters belong to a superfamily of anion transporter (Mitani-Ueno and Ma, 2021; Onuh and Miwa, 2021). Among the three subclasses (I-III) of NIPs, NIP II and III proteins respectively function as the influx channels of boric acid and silicic acid, which facilitate their permeation into the root epidermis and their xylem unloading. Unlike importers, the exporters for the xylem loading in roots and distribution in shoots of H_4_SiO_4_ and H_3_BO_3_ are the energy-dependent active efflux pumps, which have different characteristics. Si efflux transporter is a H_4_SiO_4_/H^+^ antiporter with some homology to bacterial anion transporters (Coskun et al., 2021), while B efflux transporter is the anion exchanger similar to the Cl^-^/HCO_3_^-^ transporter of animal origins (Takano et al., 2002). The Si transporters of OsLsi1 (influx) and OsLsi2 (efflux) are first identified in rice (Ma et al., 2006; Ma et al., 2007), as well as the B transporters of AtNIP5;1 (influx) and AtBOR1 (efflux) in *Arabidopsis* (Takano et al., 2002; Takano et al., 2006).

**Figure 1:**
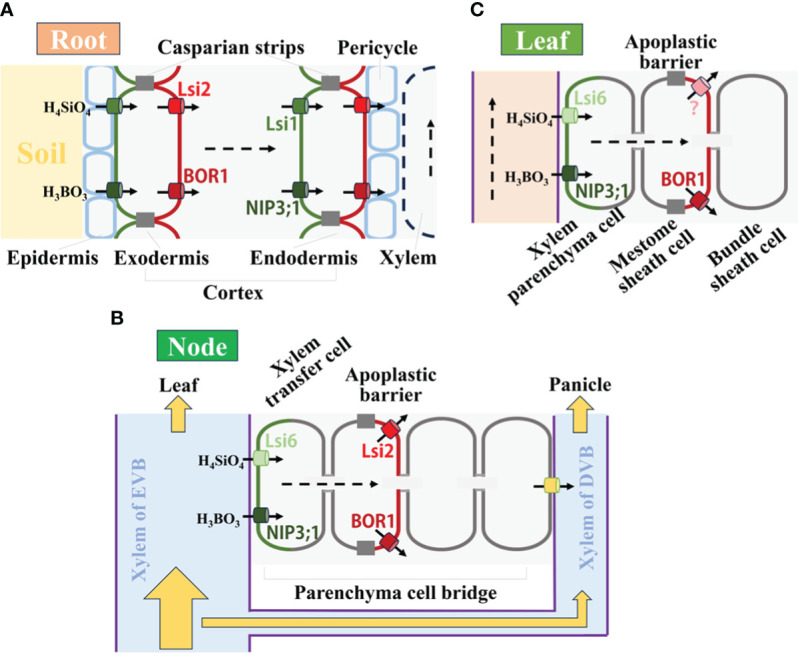
Comparable uptake and transport systems for Si and B in rice plants. **(A)** Uptake system of Si and B in roots. Two elements, in the form of H_4_SiO_4_ and H_3_BO_3_, are taken up by root using the corresponding transporters polarly localized at the distal and proximal side. **(B)** Transport system of Si and B in nodes. Si and B in the xylem of the enlarged vascular bundle are unloaded by Lsi6 and NIP3;1, respectively, which are localized at the xylem transfer cells. Then they are released toward diffuse vascular bundles by Lsi2 and BOR1. **(C)** Distributions of Si and B in leaves. After unloaded from the xylem into xylem parenchyma cells by Lsi6 and NIP3;1 respectively, B distribution is mediated by BOR1, and the transporter for further Si deposition at specific cells is an efflux pump SIET4, which polarly localizes at the distal side of epidermal cells and cells surrounding the bulliform cells.

The transport system of Si and B in plants is comparable, especially in rice plants (**Figure 1**). In roots, both Si and B importers (Lsi1 and NIP3;1) are localized to the soil side of the plasma membrane in the epidermis and endodermis (Shao et al., 2018; Konishi et al., 2023), whereas their exporters (Lsi2 and BOR1) are localized to the stele side (Shao et al., 2021; Konishi et al., 2022). The polar localization of transporters is required for the efficient uptake of two nutrient substances in the root (**Figure 1A**), especially for Si absorption. Once Si or B transport from the soil solution into the root stele is completed via the uptake system, it follows the transpiration stream and is then translocated to the shoot through the xylem (**Figures 1B, C**). In the aboveground part of plants, silicic acid will be transported from the xylem into xylem parenchyma cells or xylem transfer cells via the influx transporter Lsi6, a homolog of Lsi1(Yamaji et al., 2008). For B, NIP3;1 is functionally similar to OsLsi6 in rice plants (Shao et al., 2018). Moreover, the efflux transporters of Lsi2 and BOR1 can be expressed in shoots as well, which might be responsible for directing further Si and B transfer in rice shoots (Yamaji et al., 2015; Shao et al., 2021), respectively.

It is well known that the final distribution of Si or B in different terrestrial tissues is dependent on the tissue-specific transporters. For instance, AtNIP7;1 expressed in tapetum cells of the anthers during specific flower developmental stages, functions in the morphogenesis and germination of pollen under low-B conditions (Routray et al., 2018). Similar to AtNIP7;1, the absence of two pollen-specific transporters AtNIP4;1 and AtNIP4;2 could inhibit the pollen tube elongation and subsequent fertilization under B-limited conditions (Di Giorgio et al., 2016). These discoveries imply that tissue-specific B transporters mediate the B distribution in flowers. It was also proposed that some Si transporters specifically expressed in leaves are involved in the deposition of Si in silica cells (Mitani-Ueno et al., 2023). In rice leaf blade, an efflux Si transporter SIET4 was expressed in epidermal cells and cells surrounding the bulliform cells for Si deposition, and the knockout of the *SIET4* gene resulted in abnormal Si deposition in mesophyll cells (Mitani-Ueno et al., 2023).

So far, homologs of Si and B transporters have been identified in both monocots and dicots (Mitani-Ueno and Ma, 2021; Onuh and Miwa, 2021). In different plant species, the differences between Si or B transporters may be the expression pattern, cell-type-specific expression, and polar localization. Despite these differences, it can be roughly concluded that they form a comparable transport system for the absorption and distribution of two elements.

## 3 Chemical species of Si and B in plants

After mono-silicic acid enters the plant body and then arrives at the silicification sites, it will be deposited as the hydrated amorphous polymers (opals), which are the inorganic materials in the form of SiO_2_•nH_2_O (Epstein, 1999; Liang et al., 2015). According to their developmental patterns, biogenic plant silicas can be roughly divided into two categories: cell wall silica and cell lumen silica (Hodson, 2019; Hodson and Guppy, 2022). There is a difference in carbon and nitrogen concentrations (C/N ratios) in cell walls and lumen phytoliths (Hodson, 2019), indicating that different biomacromolecules scaffold the silica deposition. In our previous study, we also proposed that new cell wall components and silicification-related proteins serve as the kinetic drivers for promoting the silica deposition in cell walls and silica cells respectively, regardless of the effects of these biomacromolecules on the thermodynamics of silica nucleation (Sheng et al., 2023). In a word, silicon is found in plants mainly as the inorganic silica that may embed some carbohydrates or proteins within it.

However, most of B exists in the plant cell wall in the form of organoboron (B-pectin complex). A breakthrough opinion proposed by Loomis and Durst (1992) is that apiosyl residue may be the key polysaccharide moiety for the formation of B-pectin complexes. Subsequently, a boron-containing rhamnogalacturonan-II (B-RG-II) complex was purified from the root cell walls of radish plants, and the removal of boron from the complex reduces its molecular weight by half, suggesting the borate crosslinking two RG-II monomers (mRG-II) (Kobayashi et al., 1996). Furthermore, it was detected that the B-ester bonds are located on C-2 and C-3 of two 1, 3′-linked apiosyl residues of dimeric RG-II (dRG-II) (O’Neill et al., 1996). With the dRG-II-B complex isolated from the cell walls of many plant species, RG-II was considered the exclusive cell wall polysaccharide for binding boron. Additionally, in the particular plants, some soluble sorbitol–B–sorbitol, mannitol–B–mannitol, and fructose–B–fructose complexes were isolated and characterized as well (Hu et al., 1997), which may be responsible for the phloem movement of boron.

Interestingly, an obvious peak of X-ray photoelectron spectroscopy (XPS) at 101.3 ± 0.3 eV was observed in the cell wall isolated from the silicic acid-treated rice suspension cells, and this XPS peak was tentatively assigned to be Si–O–C chemical bond (He et al., 2015; Sheng et al., 2018). Further results showed that Si may link to the side chain of xyloglucan (XyG) in rice by the formation of Si–O–C bonds (Pu et al., 2021). Using the same method, the pectin-bound Si was detected in the leaf cell wall of *Dicranopteris linearis*, a Si non-accumulating plant (Zheng et al., 2023). Moreover, Si also binds to the freshly synthesized G-lignin in the root cell wall of sorghum (Zexer and Elbaum, 2020). Besides the discoveries above, the existence of Si-cell wall complexes has been identified in many plant species, including the Si-accumulating and the Si-non-accumulating plants (reviewed by Sheng and Chen, 2020). Thus, like B, a trace amount of Si was also identified as the organosilicon in the plant cell walls where wall components covalently crosslink with mono-silicic acid to form Si-ester bonds.

## 4 Similar roles of Si and B in stress responses

### 4.1 Si and B enhancement of antioxidant system for stress responses

**Figure 2:**
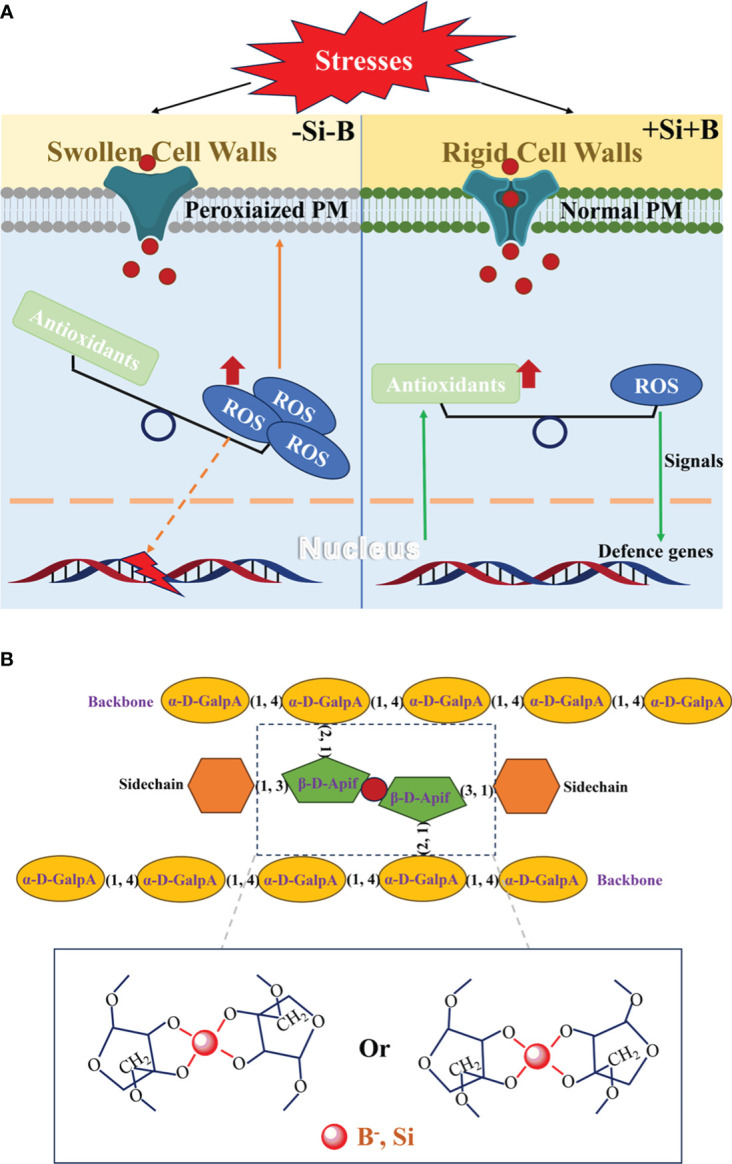
Si contributes to B in its structural function. **(A)** Similar roles of Si and B in stress responses. Si and B within the cell wall maintain the cellular ROS homeostasis under stress conditions by increasing the antioxidants, including the enzymatic and non-enzymatic antioxidants. Strengthening of cell walls and scavenging of ROS by Si and B can improve cell membrane stability by preventing the peroxidation of the plasma membrane (PM), leading to the high efficiency of transmembrane transporters. This, in turn, optimizes the acquisition of nutrients and water. Si and B also participate in the stress response in a transcription-dependent manner, which is based on the regulation of signaling molecules of ROS. In conclusion, the addition of Si and B could improve the stress resistance of plants and then promote plant growth and development under stress conditions. **(B)** The reactions of 1,3-linked apiosyl residues in RG-II with H_4_SiO_4_ and H_3_BO_3_ form the dRG-II by Si- and B-ester bonds.

It is well known that various environmental stresses can disrupt cellular homeostasis, resulting in the ROS burst in different cell compartments. A common approach for Si and B mitigating environmental stresses is the enhancement of the antioxidant defence system (**Figure 2A**), which contains the enzymatic and non-enzymatic systems. The adequate supplies of both Si and B facilitate the activation of antioxidant enzymes (such as SOD, CAT, APX, GR, and so on) under stress conditions (Hua et al., 2021), maybe in a transcription-dependent manner. In Cd-stressed rice plants, for instance, Si, B and their interactions can significantly increase the activities of SOD, CAT, and POD in the roots to alleviate Cd toxicity (Chen et al., 2019). The non-enzymatic antioxidants include the ascorbic acid (AsA), glutathione (GSH) α-tocopherol, flavonoids, carotenoids, and proline (Pro), and they can function as direct quenchers of ROS (Mostofa et al., 2021). It also has been reported that the production of some non-enzymatic antioxidants is induced by the Si and B. In cotton flowers, exogenous application of suitable-concentration Si and B can improve the contents of non-enzymatic antioxidants (carotenoids, Pro and total phenols) in petals and anthers under the B deficiency and normal conditions (de Souza Júnior et al., 2023). Overall, the acceleration of ROS scavenging by the addition of Si and B leads to an improved performance of plants under stressed conditions (Mostofa et al., 2021; Kohli et al., 2023). ROS is also a signaling molecule, therefore Si and B may trigger the signaling transduction and then reprogram the transcriptions for stress response by regulating the ROS level.

### 4.2 Stabilizing cell wall and membrane for stress resistance

On the basis of associations with silicon and boron, plant cell walls were strengthened with the changes in wall composition and organization (Zhou et al., 2017; Sheng et al., 2018). Strengthening of cell walls could improve the stress resistance for plants because the cell wall is the first barrier of defense against biotic and abiotic stresses, especially metal stress (Krzesłowska, 2011). Take aluminum (Al) stress as an example, it appears that the Si-modified cell wall is beneficial for Al deposition, thereby improving plant tolerance to aluminum toxicity. In the root apoplast, Si could ameliorate the toxic effects of Al by the formation of hydroxyaluminosilicates, being part of the mechanism (Hodson and Evans, 2020). However, Xiao et al. (2021) found that Si decreases the content of hemicellulose and increases the degree of pectin methylesterification to reduce the deposition of Al in the cell wall of root apex, thereby relieving the inhibition of root cell elongation. This suggests that the root cell wall is not the main site for Al deposition. In the shoot of many plants, thus, co-deposition of Al and Si in phytoliths, a fairly common phenomenon, may be important in the detoxification of Al (Hodson and Evans, 2020). Likewise, B supply could strengthen root cell walls with decreased pore sizes in *Citrus grandis* seedlings, thus hampering Al from getting into shoots (Tang et al., 2011). It also was reported that B modification of cell walls could enhance root elongation by decreasing Al content in roots (Li et al., 2018; Yan et al., 2022).

Si and B nutrients participate in the maintenance of cell membrane stability as well: Firstly, Si and B promotions of ROS scavenging can prevent the peroxidation of membrane systems; Secondly, rigid cell walls enhanced by the wall-bound Si and B as the structural basis stabilize cell membranes (Sheng et al., 2018). Certainly, B can directly bind to cell membranes to maintain their stability (Pollard et al., 1977). Given that cell membrane stability determines the efficiency of transmembrane transporters/channels, thus, optimization of cellular nutrient and water acquisition strategies by Si and B can substantially improve the performance in stress response (**Figure 2A**).

## 5 Why could Si partially replace B for plant growth and development?

As described above, Si can mitigate the symptoms induced by B disorder in different plants and has similar biofunctions to B in stress responses, suggesting that it can partially replace B for plant growth and development. In general, the essence of B biofunctions in plants is the borate crosslinking with dRG-II (**Figure 2B**). Moreover, turgor-driven plant cell growth is dependent on the cell wall mechanics and integrity (Sheng et al., 2018), and B deficiency causes swollen plant cell walls with decreased mechanics and integrity (Zhou et al., 2017), which impairs the process of cell division and expansion and then influences the morphogenesis of tissues and organs. It is noteworthy that both Si deposition on the cell wall and crosslinking with wall components can improve the physicochemical properties of the cell wall, including the enhancement of mechanics and integrity (Sheng and Chen, 2020). In cotton plants, Si without polymerization plays an equivalent role to B in increasing yield and fiber quality (de Souza Júnior et al., 2022). It suggests that cell wall-bound silicon, rather than silica, mainly functions as similar to boron in plant biology.

In higher plants, Si can covalently crosslink with cell wall polymers such as hemicellulose, pectin, and lignin (see above), which contain organic hydroxyl groups structurally similar to the simple *cis*-diols (Sheng and Chen, 2020). It was previously speculated that Si-cell wall complexes may form Si-ester coordinate bonds through hydroxyl complexation between mono-silicic acid and *cis*-diols (Sheng and Chen, 2020), with the reaction rationale like the formation of B-ester bonds by formose reaction (He et al., 2015). Particularly, Si may replace B to bind the apiosyl residues of dRG-II, thereby forming the Si-pectin complex (**Figure 2B**; Sheng and Chen, 2020). Therefore, herein we conclude that silicon may partially substitute for boron in plant biology, due to the Si contribution to B in its structural function (**Figure 2B**).

## 6 Conclusions and future perspectives

Two neighboring metalloid elements of Si and B are crucial for plant growth and development, as well as stress tolerance. Plants have evolved comparable transport systems to absorb these two elements in the form of H_4_SiO_4_ and H_3_BO_3_, arising from substrates with similar chemical characteristics. Regardless of the Si polymerization to inorganic silica, both silicic acid and boric acid form complexes with cell wall components in higher plants, whose processes depend on the hydroxyl complexation between them and ligands. The additions of Si and B reinforce cell walls, scavenge ROS, and stabilize cell membranes. This, in turn, promotes nutrient uptake and improves the performance of plants in terms of growth, metabolism, and stress resistance. Based on the analogy found in the chemical and biological characters of two elements and Si alleviation of B deficiency, it can be concluded that Si might partially substitute for boron in plant nutrition because of the Si contribution to B in its structural function.

To date, whether the formation of the dRG-II-Si complex replaces the dRG-II-B complex under the B-limited conditions in plants is uncertain, it needs detailed investigations in the future. RG-II dimers/monomers can be isolated and quantified using an evaporative light-scattering detector (ELSD) (Zhou et al., 2017). In this case, increased RG-II dimers in the Si-modified cell wall suggest the formation of dRG-II-Si complex. Subsequently, the detection of the Si–O–C bond by XPS should be performed in the isolated RG-II dimers. These results are solid evidence for the presence or absence of dRG-II-Si complex. Overall, these efforts will help improve our understanding of the functional analogy of silicon and boron in plant nutrition.

## Author contributions

HS: Conceptualization, Funding acquisition, Writing – original draft, Writing – review & editing. YYL: Data curation, Visualization, Writing – review & editing. JW: Data curation, Visualization, Writing – review & editing. ZY: Investigation, Software, Writing – review & editing. LP: Project administration, Validation, Writing – review & editing. WL: Conceptualization, Validation, Writing – original draft, Writing – review & editing. YL: Conceptualization, Supervision, Writing – original draft, Writing – review & editing.
